# Screen-detected and interval breast cancer after concordant and discordant interpretations in a population based screening program using independent double reading

**DOI:** 10.1007/s00330-022-08711-9

**Published:** 2022-04-02

**Authors:** Marit A. Martiniussen, Silje Sagstad, Marthe Larsen, Anne Sofie F. Larsen, Tone Hovda, Christoph I. Lee, Solveig Hofvind

**Affiliations:** 1 Department of Radiology, Østfold Hospital Trust, Kalnes, Norway; 2 University of Oslo, Institute of Clinical Medicine, Oslo, Norway; 3 Section for Breast Cancer Screening, Cancer Registry of Norway, P.O. Box 5313, 0304 Oslo, Norway; 4 Department of Radiology, Vestre Viken Hospital Trust, Drammen, Norway; 5 Department of Radiology, University of Washington School of Medicine, Seattle, Washington USA; 6 Department of Health Systems and Population Health, University of Washington School of Public Health, Seattle, Washington USA; 7 Department of Health and Care Sciences, UiT The Artic University of Norway, Tromsø, Norway

**Keywords:** Breast neoplasms, Mammography, Mass screening, Consensus, Interval cancer

## Abstract

**Objectives:**

To analyze rates, odds ratios (OR), and characteristics of screen-detected and interval cancers after concordant and discordant initial interpretations and consensus in a population-based screening program.

**Methods:**

Data were extracted from the Cancer Registry of Norway for 487,118 women who participated in BreastScreen Norway, 2006–2017, with 2 years of follow-up. All mammograms were independently interpreted by two radiologists, using a score from 1 (negative) to 5 (high suspicion of cancer). A score of 2+ by one of the two radiologists was defined as discordant and 2+ by both radiologists as concordant positive. Consensus was performed on all discordant and concordant positive, with decisions of recall for further assessment or dismiss. OR was estimated with logistic regression with 95% confidence interval (CI), and histopathological tumor characteristics were analyzed for screen-detected and interval cancer.

**Results:**

Among screen-detected cancers, 23.0% (697/3024) had discordant scores, while 12.8% (117/911) of the interval cancers were dismissed at index screening. Adjusted OR was 2.4 (95% CI: 1.9–2.9) for interval cancer and 2.8 (95% CI: 2.5–3.2) for subsequent screen-detected cancer for women dismissed at consensus compared to women with concordant negative scores. We found 3.4% (4/117) of the interval cancers diagnosed after being dismissed to be DCIS, compared to 20.3% (12/59) of those with false-positive result after index screening.

**Conclusion:**

Twenty-three percent of the screen-detected cancers was scored negative by one of the two radiologists. A higher odds of interval and subsequent screen-detected cancer was observed among women dismissed at consensus compared to concordant negative scores. Our findings indicate a benefit of personalized follow-up.

**Key Points:**

*• In this study of 487,118 women participating in a screening program using independent double reading with consensus, 23% screen-detected cancers were detected by only one of the two radiologists.*

*• The adjusted odds ratio for interval cancer was 2.4 (95% confidence interval: 1.9, 2.9) for cases dismissed at consensus using concordant negative interpretations as the reference.*

*• Interval cancers diagnosed after being dismissed at consensus or after concordant negative scores had clinically less favorable prognostic tumor characteristics compared to those diagnosed after false-positive results.*

**Supplementary Information:**

The online version contains supplementary material available at 10.1007/s00330-022-08711-9.

## Introduction

Mammographic screening is shown to reduce mortality from breast cancer and is recommended by international health organizations [1, 2]. However, the identification of asymptomatic breast cancers presenting as subtle mammographic findings are challenging, with 20–25% of interval cancers reported to be visible at prior mammograms in informed reviews [3]. Studies from Europe have shown that double reading increased the rate of screen-detected cancer [4]. The recall rate has been shown to be higher for double reading without consensus or arbitration meeting [5], but lower if double reading was followed by consensus or arbitration meeting [6], compared with single reading. European guidelines and the European Commission Initiative on Breast Cancer suggest double reading with consensus or arbitration, but do not specify if double reading should be independent or not [1, 7].

Women with false-positive screening results in double-reading programs are shown to have increased risk of interval cancer and cancer detected in the consecutive screening round [8]. However, less is known about the risk of interval cancer among women with screening examinations discussed and dismissed at consensus as well as the prognostic characteristics of such tumors. Two studies have reported a higher interval cancer rate after being dismissed at consensus compared to those with concordant negative screening results [9, 10]. To examine this, we obtained data collected as a part of BreastScreen Norway, which provides detailed information about the radiologists’ interpretation at both initial screening and consensus, as well as the screening outcome. In this study, we aimed to analyze the odds of screen-detected, interval, and subsequent screen-detected cancer by initial interpretation scores and consensus. Furthermore, we described differences in histopathologic tumor characteristics by screening and consensus interpretations.

## Materials and methods

This retrospective cohort study was approved by the data protection official at Oslo University Hospital (20/12601). The data was disclosed with legal bases in the Norwegian Cancer Registry Regulations of 21 December 2001 No. 47 [11].

BreastScreen Norway is a population-based screening program which started in 1996 and invites all women aged 50–69 to biennial two-view mammography. The program is described in detail elsewhere [12]. Briefly, the Cancer Registry of Norway administers the program and collects information about screening examinations, recalls, diagnostic work-ups, treatment, and surveillance. Digital mammography replaced screen-film mammography gradually from 2004, and all women have been offered digital mammography since 2011. During the first 20 years of the screening program, the annual participation rate in the screening program was 75%, the consensus rate 7%, and the recall rate 3.8%. The rate of screen-detected cancer was 5.9 per 1000 screening examinations and the interval cancer rate 1.8 per 1000 examinations.

Independent double reading with consensus is standard practice in BreastScreen Norway. Each breast is assigned a score from 1 to 5 by each radiologist, where 1 indicates normal findings; 2 probably benign; 3 intermediate suspicion; 4 probably malignant; and 5 high suspicion of malignancy. If both radiologists give a score of 1, the screening examination is considered negative. If either radiologist assigns a score of 2 or higher for one or both breasts, the exam is discussed in consensus to determine whether to recall the woman for further assessment (recall) or not (dismiss). If consensus is not met by the two radiologists, a third is consulted. Examinations dismissed at consensus are considered screen-negative. We defined discordant interpretation as a score of 1 by one of two radiologists and 2 or higher by the other. A score of 2 or higher by both radiologists was defined as concordant positive, while a score of 1 by both radiologists was considered concordant negative. During the study period, 2006–2019, 196 radiologists were registered as readers in the program. The median annual average interpretations per radiologist were 2992 examinations (interquartile range (IQR): 1357–5327).

**Fig. 1:**
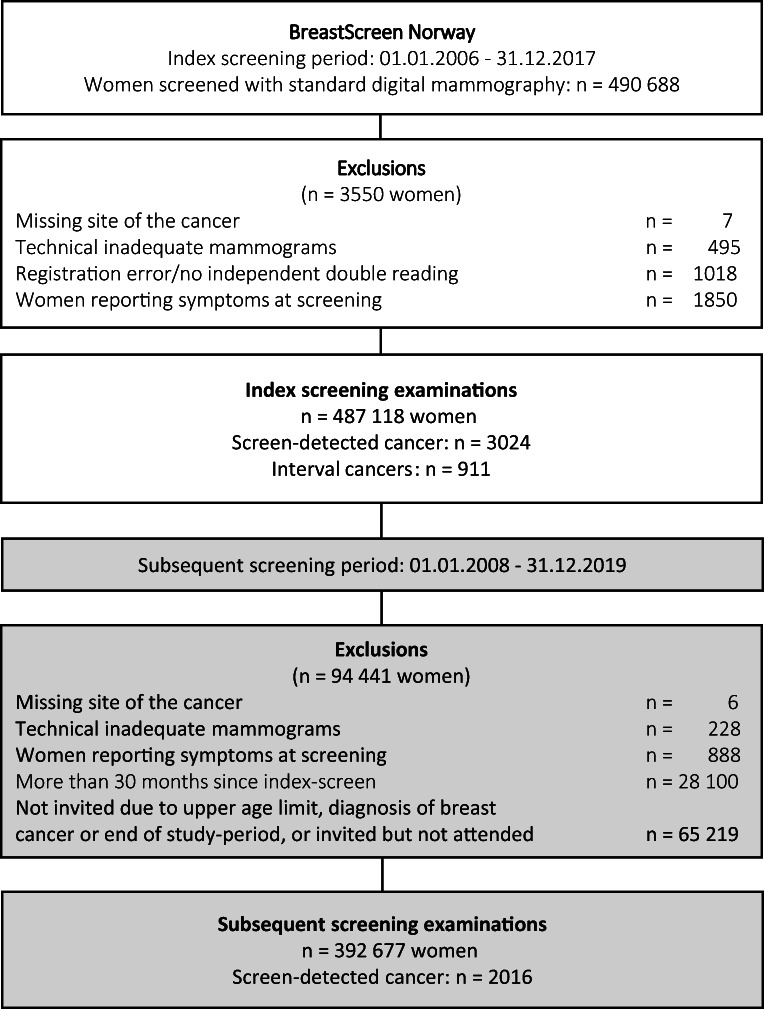
Flowchart of the study population. Reasons for exclusions, number of index study population and subsequent study population, number of screen-detected cancers, interval cancers, and subsequent screen-detected cancers

**Fig. 2:**
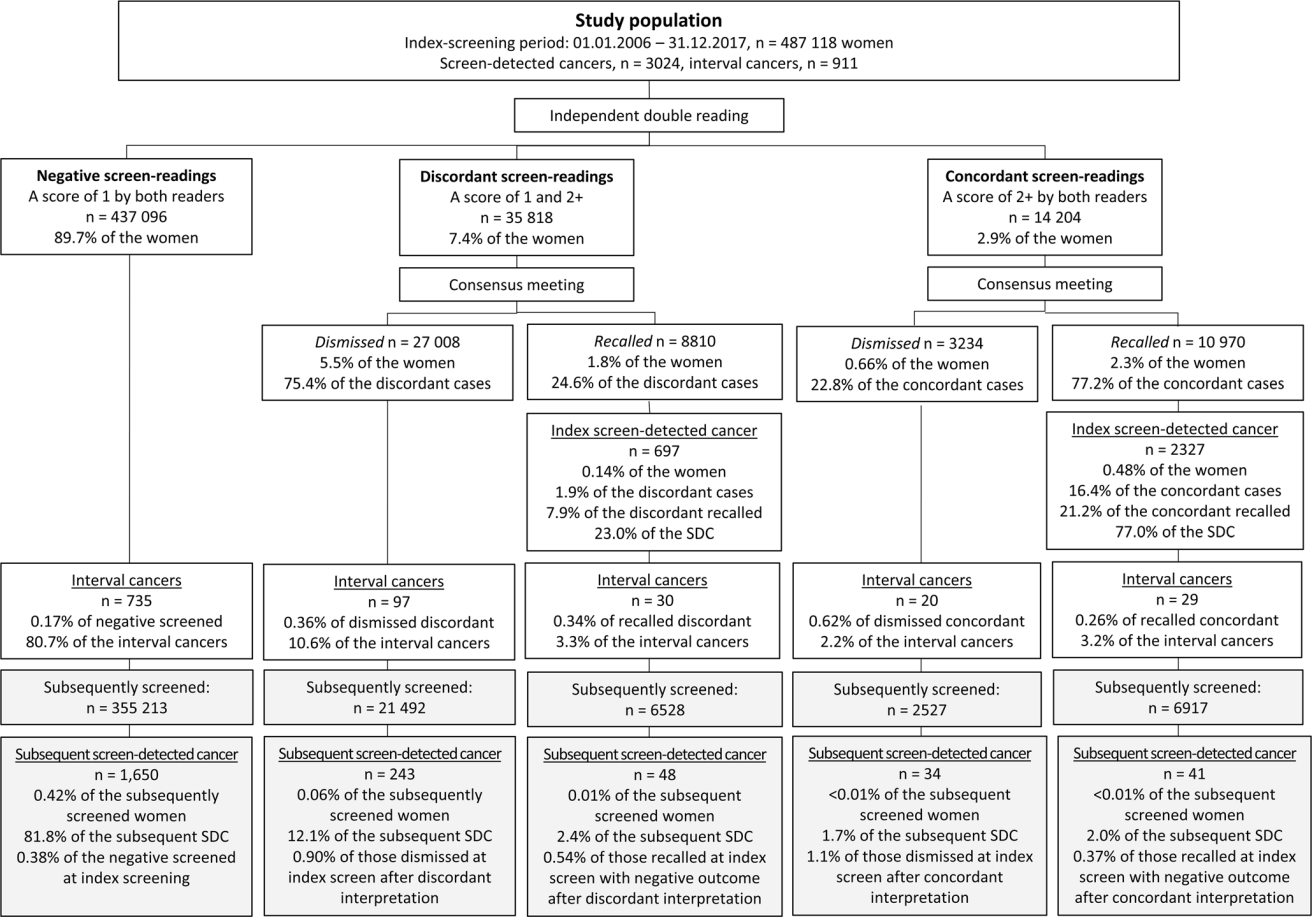
Flowchart of number of screening mammograms stratified by results of interpretation score at index screen and outcome of consensus. Recall rates, cancer detection rate, proportion of discordant and concordant cancers, and number of interval cancers and subsequent screen-detected cancers, in a population-based screening program using independent double reading with consensus

The study sample included women without a history of breast cancer, screened with standard digital mammography within the study period. To ensure availability of prior digital mammograms for comparison at the time of interpretation, the study period started 2 years after implementation of digital mammography at the 17 centers in BreastScreen Norway. The women were followed for 2 years after index screen to identify interval and screen-detected cancers in the consecutive screening round. Index screenings were performed in 2006–2017, while subsequent screenings were performed in 2008–2019 (Fig. 1). Index screenings included women who had their first screening (prevalent) and women with a previous screening (incident) in BreastScreen Norway (Appendix, Figure 1 and 2). We excluded mammograms that were technically inadequate (*n* = 495), those with registration error or no independent double reading (*n* = 1018), and those performed among women with self-reported symptoms (*n* = 1850).

A screen-detected cancer was defined as breast cancer (ductal carcinoma in situ (DCIS) or invasive breast cancer) diagnosed after a recall. If a recall was concluded negative within 6 months after screening, the screening result was considered false positive. Interval cancer was defined as breast cancer detected after a negative screening result or more than 6 months after a false-positive screening result and within 24 months after screening [8]. For women diagnosed with two or more bilateral synchronous breast tumors, we included the interpretation scores from the breast with the highest score.

Histopathologic tumor characteristics were based on surgical specimens and included histologic type (DCIS, invasive carcinoma no special type, invasive lobular carcinoma, and other types of invasive carcinomas), tumor diameter (mm), histologic grade (grade 1–3), and lymph node involvement. Immunohistochemical subtypes were based on estrogen receptor (ER), progesterone receptor (PR), and human epidermal growth factor receptor 2 (Her2) status, given as Luminal A–like, Luminal B–like Her2−, Luminal B–like Her2+, Her2+, and triple negative [13].

### Statistical analysis

All analyses were conducted at the woman level rather than at breast level to ensure clinical applicable results. We stratified the index screening examinations by negative, discordant, and concordant positive scores and further into dismissed and recalled at consensus.

We used logistic regression to estimate odds of index screen-detected, interval, and incident screen-detected cancer. Results were presented as ORs with 95% confidence intervals (CIs), adjusted for age and prevalent/incident screenings. Chi-square or Fisher exact test was used to test associations between categorical variables (tumor characteristics) and discordant and concordant positive scores, or negative, dismissed, and false-positive screening results. We used the non-parametric test for comparing tumor diameters. A significance level of 0.05 was chosen, and all statistical analyses were performed with Stata MP Version 17.0 (StataCorp).

## Results

We obtained data about radiologist interpretation scores, consensus, recall, cancer diagnosis, and histopathological tumor characteristics for 490,688 index screened women (Fig. 1). After exclusions, data on 487,118 women were available, 184,736 prevalent screenings (Appendix, Figure 1) and 302 382 incident screenings (Appendix, Figure 2). After exclusions, 392,677 women were followed until the subsequent screening, 2 years later (Fig. 1).

### Recall rate, cancer detection rate, and rate of discordant cancers

Independent double reading resulted in a recall rate of 4.1% (19, 780/487,118), 1.8% (8810/487,118) due to discordant and 2.3% (10, 970/487,118) due to concordant positive scores (Fig. 2). At index screening, the rate of screen-detected cancer was 0.62% (3024/487,118), 0.14% (697/487,118) after discordant, and 0.48% (2327/487,118) after concordant positive scores, which means that discordant cases made up 23.0% (697/3024) of index screen-detected cancer. Adjusted OR for screen-detected cancer was 11.6 (95% CI: 10.6, 12.7) for cases with concordant versus discordant scores (Table 1). The rate of interval cancer was 0.19% (911/487,118). Among women with interval cancer, 12.8% (117/911) were dismissed at index screening, 6.5% (59/911) had a false-positive screening result, and 80.7% (735/911) had concordant negative scores (Fig. 2).

**Table 1:** Crude and adjusted odds ratios with 95% confidence intervals (CIs) of screen-detected, interval, and subsequent screen-detected cancers in BreastScreen Norway. The exposure variables (interpretation score and outcome of consensus) were modeled separately. The adjusted models accounted for age and prevalent/incident attendance

	Crude			Adjusted		
	Odds ratio	95% CI	*p* value	Odds ratio	95% CI	*p* value
Screen-detected cancer			< 0.001			< 0.001
Discordant	Reference			Reference		
Concordant positive	9.9	9.1, 10.8		11.6	10.6, 12.7	
Interval cancer			< 0.001			< 0.001
Concordant negative	Reference			Reference		
Discordant	2.1	1.8, 2.6		2.2	1.8, 2.6	
Concordant positive	2.1	1.5, 2.8		2.1	1.6, 2.8	
Outcome of consensus at index screen			< 0.001			< 0.001
Concordant negative	Reference			Reference		
Dismissed	2.3	1.9, 2.8		2.4	1.9, 2.9	
False-positive screening result	1.8	1.4, 2.3		1.8	1.4, 2.4	
Subsequent screen-detected cancer			< 0.001			< 0.001
Concordant negative	Reference			Reference		
Discordant	2.3	2.0, 2.6		2.6	2.3, 2.9	
Concordant positive	1.7	1.4, 2.2		2.1	1.6, 2.6	
Outcome of consensus at index screen			< 0.001			< 0.001
Concordant negative	Reference			Reference		
Dismissed	2.5	2.2, 2.8		2.8	2.5, 3.2	
False-positive screening result	1.4	1.2, 1.8		1.7	1.4, 2.1	

Among examinations dismissed at consensus, 89.3% (27,008/30,242) had discordant scores. Adjusted OR for interval cancer was 2.4 (95% CI: 1.9, 2.9) for those dismissed at index screening and 1.8 (95% CI: 1.4, 2.4) for those with a false-positive screening examination, using concordant negative scores as reference (Table 1). The rate of subsequent screen-detected cancer was 0.51% (2016/392,677), where 13.7% (277/2016) were dismissed at index screening, 4.4% (89/2016) were false-positive, and 81.8% (1650/2016) were concordant negative at index screening (Fig. 2). Using concordant negative as reference, adjusted OR for subsequent screen-detected cancer was 2.8 (95% CI: 2.5, 3.2) for those dismissed at index screening and 1.7 (95% CI: 1.4, 2.1) for those with a false-positive screening result (Table 1).

**Fig. 3:**
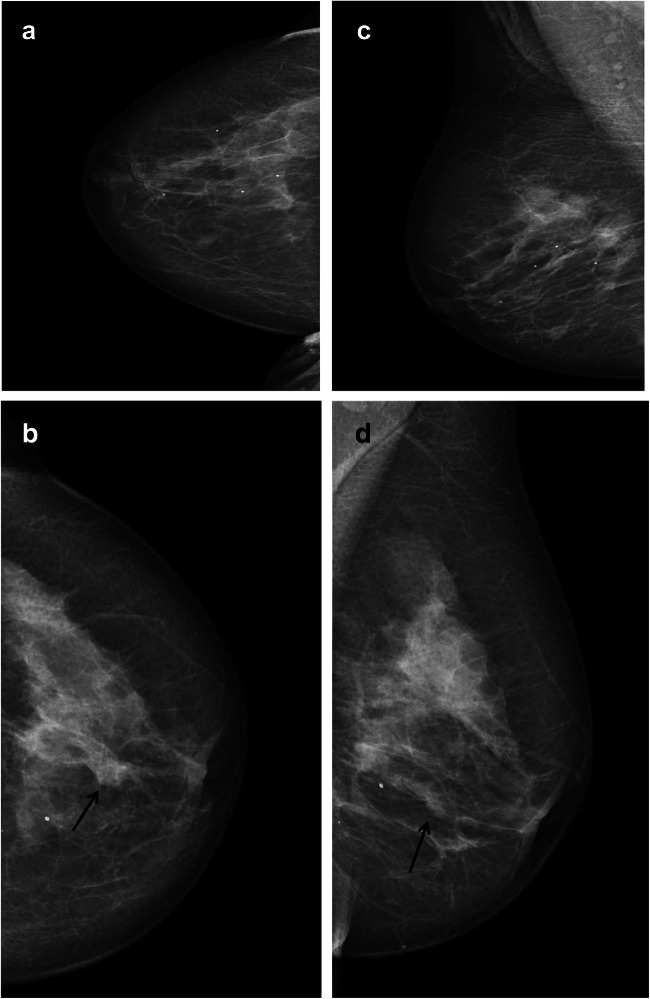
Craniocaudal (**a** and **b**) and mediolateral oblique (**c** and **d**) mammograms of both breasts from a 69-year-old woman diagnosed with screen-detected cancer after discordant score. The cancer presented as an asymmetry of the left breast (arrows)

**Fig. 4:**
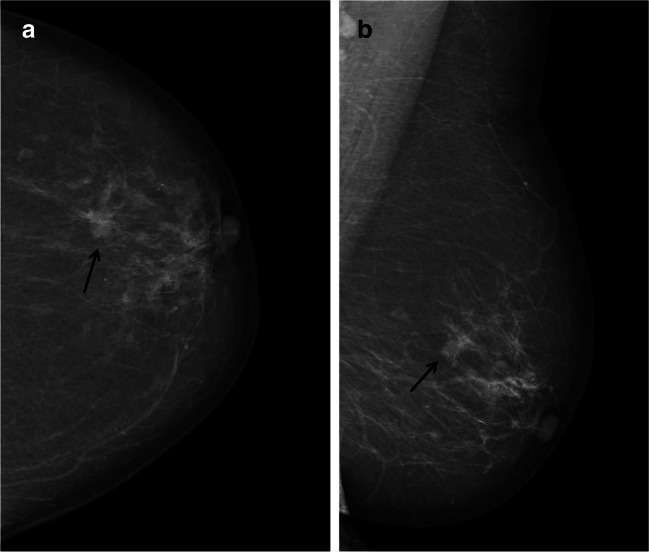
Craniocaudal (**a**) and mediolateral oblique (**b**) mammograms of the left breast in a 67-year-old woman diagnosed with screen-detected cancer after concordant score. The cancer presented as a small spiculated mass in the upper lateral quadrant (arrow)

Recall rate was 7.0% (12,891/184,736) for prevalent and 2.3% (6889/302,382) for incident screenings (Appendix, Figure 3 and 4). The rate of screen-detected cancer was 0.68% (1253/184,736) for prevalent and 0.59% (1771/302,382) for incident screenings, while the rate of interval cancer was 0.19% both for prevalent (349/184,736) and incident screening examinations (562/302,382). The proportion of discordant screen-detected cancers was 20.0% (250/1253) for prevalent and 25.2% (447/1771) for incident screening examinations. Among prevalent screening examinations, 12.3% (43/349) of interval cancers and 16.0% (92/576) of subsequent screen-detected cancers were dismissed at consensus after discordant scores. Among incident screening examinations, 9.6% (54/562) of the interval cancers and 10.5% (151/1440) of the subsequent screen-detected cancers were dismissed after discordant scores.

### Histopathological tumor characteristics

For screen-detected cancers, the proportion of DCIS was 25.3% (176/697) for discordant and 16.2% (377/2327) for concordant positive cases (Table 2). Median tumor diameter was 11 mm (IQR: 8–17 mm) for discordant and 14 mm (IQR: 9–20 mm) for concordant positive cancers, while the proportion of lymph node involvement was 16.4% (83/505) versus 23.6% (451/1914), respectively. Luminal A–like immunohistochemical subtype accounted for 69.0% (310/449) of the discordant and 62.1% (1048/1687) of the concordant positive screen-detected cancers.

**Table 2:** Tumor characteristics of index screen-detected cancers, stratified by discordant and concordant scores in BreastScreen Norway

	All (*n* = 3024)	Discordant scores (*n* = 697)	Concordant positive scores (*n* = 2327)	*p* value^*^
Histopathological characteristics				
Histopathological type				< 0.001
Ductal carcinoma in situ	553 (18.3)	176 (25.3)	377 (16.2)	
Invasive carcinoma of NST	2106 (69.6)	433 (62.1)	1673 (71.9)	
Invasive lobular carcinoma	240 (7.9)	64 (9.2)	176 (7.6)	
Other invasive carcinoma	125 (4.1)	24 (3.4)	101 (4.3)	
Invasive tumors	2471 (81.7)	521 (74.8)	1950 (83.8)	< 0.001
Tumor diameter, median (IQR), mm	13 (9–20)	11 (8 – 17)	14 (9 – 20)	< 0.001
Data not available	52	9	43	
Histological grade				< 0.001
Grade 1	752 (30.8)	195 (38.0)	557 (28.9)	
Grade 2	1163 (47.6)	251 (48.9)	912 (47.3)	
Grade 3	528 (21.6)	67 (13.1)	461 (23.9)	
Data not available	28	8	20	
Lymph node positive	534 (22.1)	83 (16.4)	451 (23.6)	0.001
Data not available	52	16	36	
Immunohistochemical subtypes				0.072
Luminal A–like (ER+/PR+/Her2−)	1358 (63.6)	310 (69.0)	1048 (62.1)	
Luminal B–like Her2− (ER+/PR−/Her2−)	311 (14.6)	57 (12.7)	254 (15.1)	
Luminal B–like Her2+ (ER+/PR±/Her2+)	264 (12.4)	44 (9.8)	220 (13.0)	
Her2+ (ER−/PR−/Her2+)	87 (4.1)	19 (4.2)	68 (4.0)	
Triple negative (ER−/PR−/Her2−)	116 (5.4)	19 (4.2)	97 (5.7)	
Data not available	335	72	263	

Unless otherwise specified, data are presented as numbers with percentage in parenthesis

*IQR* interquartile range, *NST* no special type, *ER* estrogen receptor, *PR* progesterone receptor, *Her2* human epidermal growth factor receptor

^*^Overall *p* value for association between discordant/concordant scores and the different tumor characteristics

The proportion of interval cancers that were DCIS was 3.7% (27/735) in concordant negative cases, 3.4% (4/117) in dismissed cases, and 20.3% (12/59) for women screened false-positive at index screening (Table 3). Median tumor diameter was 19 mm (IQR: 13–26 mm) in concordant negative cases, 20 mm (IQR: 14–29 mm) in dismissed cases, and 15 mm (IQR: 11–22 mm) in cases with false positive index screening (*p* value 0.089). Lymph node positive cancers were 39.9% (268/672) in concordant negative cases, 42.1% (45/107) in dismissed cases, and 27.9% (12/43) in cases with false-positive index screening (*p* value 0.248).

**Table 3:** Tumor characteristics of interval cancers, stratified by negative index screening, dismissed at index screening, and false-positive screening results in BreastScreen Norway

	All (*n* = 911)	Concordant negative (*n* = 735)	Dismissed (*n* = 117)	False-positive screening result (*n* = 59)	*p* value*
Histopathological type					< 0.001
Ductal carcinoma in situ	43 (4.7)	27 (3.7)	4 (3.4)	12 (20.3)	
Invasive carcinoma NST	716 (78.6)	584 (79.5)	97 (82.9)	35 (59.3)	
Invasive lobular carcinoma	122 (13.4)	99 (13.5)	13 (11.1)	10 (17.0)	
Other invasive cancers	30 (3.3)	25 (3.4)	3 (2.6)	2 (3.4)	
Invasive tumors	868 (95.3)	708 (96.3)	113 (96.6)	47 (79.7)	< 0.001
Tumor diameter median (IQR), mm	19 (13–26)	19 (13–26)	20 (14–29)	15 (11–22)	0.089
Data not available	77	64	9	4	
Histological grade					0.192
Grade 1	97 (11.6)	76 (11.1)	12 (11.2)	9 (20.0)	
Grade 2	386 (46.2)	325 (47.5)	42 (39.3)	19 (42.2)	
Grade 3	353 (42.2)	283 (41.4)	53 (49.5)	17 (37.8)	
Data not available	32	24	6	2	
Lymph node positive	325 (39.5)	268 (39.9)	45 (42.1)	12 (27.9)	0.248
Data not available	45	36	6	4	
Immunohistochemical subtypes					0.286
Luminal A–like (ER+/PR+/Her2−)	355 (46.5)	1286 (46.4)	145 (43.7)	24 (55.8)	
Luminal B–like Her2− (ER+/PR−/Her2−)	115 (15.1)	90 (14.6)	16 (15.5)	9 (20.9)	
Luminal B–like Her2+ (ER+/PR±/Her2+)	140 (18.3)	111 (18.0)	26 (25.2)	3 (7.0)	
Her2+ (ER−/PR−/Her2+)	48 (6.3)	42 (6.8)	4 (3.9)	2 (4.7)	
Triple negative (ER−/PR−/Her2−)	105 (13.8)	88 (14.3)	12 (11.7)	5 (11.6)	
Data not available	105	91	10	4	

Unless otherwise specified, data are presented as numbers with percentage in parenthesis

*IQR* interquartile range, *NST* no special type, *ER* estrogen receptor, *PR* progesterone receptor, *Her2* human epidermal growth factor receptor

^*^Overall *p* value for association between concordant negative/dismissed/false-positive screening results, and the different tumor characteristics

Among subsequent screen-detected cancers, the histopathological characteristics did not differ significantly based on consensus outcome. The proportion of DCIS was 17.4% (287/1650) for concordant negative cases, 18.1% (50/277) for dismissed cases, and 21.3% (19/89) for women with a false-positive index screening (Table 4). For invasive cancers, the proportion of Luminal A–like immunohistochemical subtype was 59.6% (759/1274) for concordant negative cases, 69.2% (144/208) for dismissed cases, and 64.5% (40/62) for false-positive cases at index screening.

**Table 4:** Tumor characteristics of subsequent screen-detected cancer, stratified by negative index screening, dismissed at index screening, and false-positive screening results in BreastScreen Norway

	All (*n* = 2016)	Concordant negative (*n* = 1650)	Dismissed (*n* = 277)	False-positive screening result (*n* = 89)	*p* value*
Histopatological type					0.529
Ductal carcinoma in situ	356 (17.7)	287 (17.4)	50 (18.1)	19 (21.3)	
Invasive carcinoma NST	1 417 (70.3)	1 172 (71.0)	187 (67.5)	58 (65.2)	
Invasive lobular carcinoma	169 (8.4)	135 (8.2)	25 (9.0)	9 (10.1)	
Other invasive cancers	74 (3.7)	56 (3.4)	15 (5.4)	3 (3.4)	
Invasive tumors	1 660 (82.3)	1 363 (82.6)	227 (82.0)	70 (78.7)	0.624
Tumor diameter median (IQR), mm	13 (9–19)	13 (9–19)	14 (9–20)	13 (10–21)	0.772
Data not available	26	20	4	2	
Histological grade					0.295
Grade 1	441 (26.8)	371 (27.4)	58 (25.9)	12 (17.4)	
Grade 2	836 (50.8)	674 (49.9)	120 (53.6)	42 (60.9)	
Grade 3	368 (22.3)	307 (22.7)	46 (20.5)	15 (21.7)	
Data not available	15	11	3	1	
Lymph node positive	307 (18.8)	250 (18.6)	44 (19.6)	13 (18.8)	0.934
Data not available	23	19	3	1	
Immunohistochemical subtypes					0.175
Luminal A–like (ER+/PR+/Her2−)	943 (61.1)	759 (59.6)	144 (69.2)	40 (64.5)	
Luminal B–like Her2− (ER+/PR−/Her2−)	183 (11.9)	155 (12.2)	21 (10.1)	7 (11.3)	
Luminal B–like Her2+(ER+/PR±/Her2+)	285 (18.5)	242 (19.0)	33 (15.9)	10 (16.1)	
Her2+ (ER−/PR−/Her2+)	47 (3.0)	39 (3.1)	6 (2.9)	2 (3.2)	
Triple negative (ER−/PR−/Her2−)	86 (5.6)	79 (6.2)	4 (1.9)	3 (4.8)	
Data not available	116	89	19	8	

Unless otherwise specified, data are presented as numbers with percentage in parenthesis

*IQR* interquartile range, *NST* no special type, *ER* estrogen receptor, *PR* progesterone receptor, *Her2* human epidermal growth factor receptor

^*^Overall *p* value for association between discordant/discordant scores and the different tumor characteristics

## Discussion

**Fig. 5:**
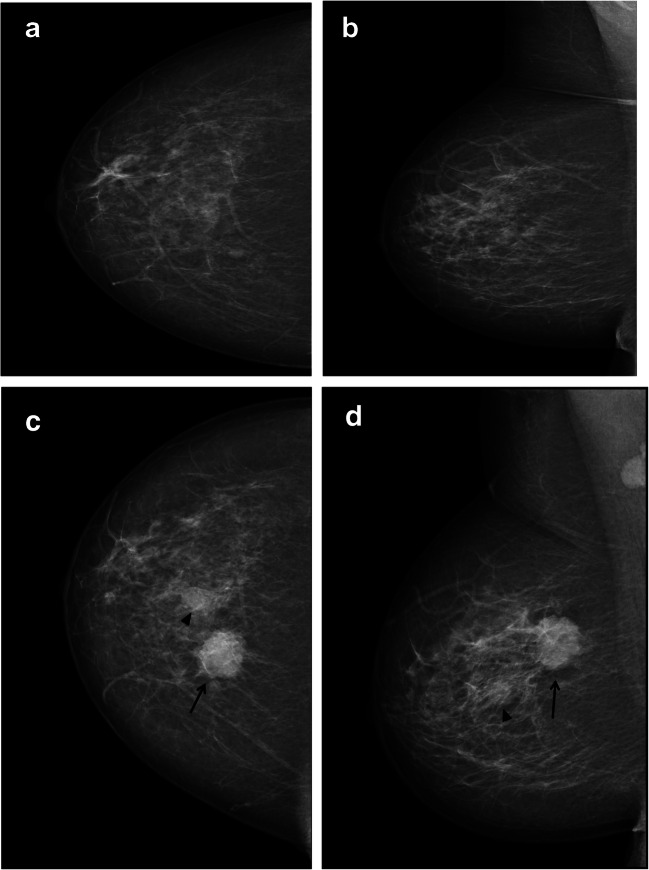
The craniocaudal and mediolateral oblique mammograms of the right breast at index (**a** and **b**) and subsequent screening (**c** and **d**) from a 54-year-old woman diagnosed with subsequent screen-detected cancer after false-positive index screening. The examination was characterized as a one-plane asymmetry in the craniocaudal view at index screening. At subsequent screening, a circumscribed mass in the upper medial quadrant (arrow) and a smaller mass, located more lateral and inferior (arrowhead), were both histologically verified as cancers

We found that nearly a quarter (23%) of screen-detected cancers were scored negative by one of two interpreting radiologists in an organized screening program using independent double reading with consensus (Figs. 3, 4, and 5). Examinations discussed and dismissed at consensus had higher odds of interval and subsequent screen-detected cancer compared to concordant negative examinations. Histopathological results indicate that interval cancers diagnosed after being dismissed at consensus or after concordant negative scores had less favorable prognostic histopathologic tumor characteristics compared to those diagnosed after a false-positive screening result.

Our results showing higher odds of interval cancer after being dismissed at consensus are in line with previous studies [9, 10]. A study from UK reported the rate to range from 6.1 to 7.7/1000 screening examinations, while results from a Norwegian study ranged from 2.9 to 3.1/1000. For comparison, the rates for negatively screened were 2.9/1000 screening examinations in the UK and 1.7/1000 in Norway.

A lower proportion of discordant screen-detected cancers was observed among prevalent (20.0%) versus incident (25.2%) screened women. This was also observed in a previous study from BreastScreen Norway, using mainly analog mammograms [10]. Screen-detected cancers among incident screened women have been associated with a smaller proportion of advanced breast cancer compared to first-time, prevalent screened women [14]. However, histopathological tumor size has been reported to be similar among prevalent and incident screenings [12]. Future studies focusing on comparing tumor characteristics between these two groups would help fill this knowledge gap.

In this study, 7.4% of all screening examinations were discussed at consensus due to discordant scores and 75.4% of these were dismissed at consensus. We found that 10.6% of interval cancers and 12.1% of subsequent screen-detected cancers were discordant cases discussed and dismissed at consensus. In other words, 340 (1.3%) out of the 27,008 women with dismissed examinations were diagnosed with breast cancer within 2 years. Using a 1-year follow-up strategy for discordant cases dismissed at consensus may be one strategy for lowering the interval cancer rate and increasing the rate of screen-detected cancer. However, this may also increase the recall rate and false-positive screening rates and increase workload for radiologists. Use of tomosynthesis represents a possible strategy due to the higher rate of screen-detected cancers [15–17]. However, there are variable results on recall, interval cancer, and reading time compared to standard digital mammography. Formal cost-effectiveness analyses would help weight the benefits versus costs of such approaches. Another strategy could be use of artificial intelligence (AI). AI has the potential to increase the accuracy of screening interpretations and reduce the radiologists’ workload, costs, and subjectivity of the interpretation. Studies introducing AI in the reading process have shown promising results with some studies reporting performance at the level of radiologists [18, 19]. However, so far, the evidence is scarce due to small study populations, enhanced data sets often used to train the models, and lack of prospective studies [20, 21].

Our findings of prognostic favorable histopathological tumor characteristics for discordant screen-detected cancers versus concordant positive cases are consistent with other studies [5, 6]. For interval cancers diagnosed after being dismissed at consensus, the rate of invasive cancers was higher among dismissed and concordant negative compared to false-positive cases. Although not significantly different, the results of more lymph node involvement, a lower proportion of histological grade 1 invasive cancers and Luminal A–like immunohistochemical subtype among dismissed and concordant negative examinations indicates less favorable prognostic characteristics compared to cancers detected after false-positive screening.

High completeness of the data and detailed information about the radiologist’s interpretation scores represent strengths of this study. However, despite a large study population, some subgroups had few cancer cases resulting in less powerful results. Using woman-level rather than breast-level analyses ensures the clinical approach, on the cost of the accuracy as some of the cancers might be in the other breast than the positive score at index screening. Further, some features that resulted in a positive score at index screening might not be the same as later diagnosed as cancer, even though they appeared in the same breast. A previous retrospective review of screening mammograms in BreastScreen Norway identified that 42.9% of interval cancers diagnosed after a false-positive screening were recalled due to the same mammographic finding [8]. Further, the scoring system used in BreastScreen Norway represents a modified version of BI-RADS [22]. A score of 1 in the Norwegian system corresponds to BI-RADS 1 and 2, scores 2, 3, 4, and 5 are analog to BI-RADS 3, 4a–b, 4c, and 5, respectively, while BI-RADS 0 and 6 do not apply. We consider these differences not affecting the generalizability of our study.

In conclusion, 23% of screen-detected cancers were detected by only one of two radiologists. The odds of interval and subsequent screen-detected cancer was 2–3 times higher for women with examinations discussed but dismissed at consensus for index screening compared to those with concordant negative scores. Adding an additional screening 1 year after being dismissed at consensus or exploiting AI in screen-reading and at the time of consensus are potential strategies that may be considered for the purpose of reducing interval cancers.

## Supplementary Information

ESM 1 (DOCX 359 kb)

## Abbreviations

AI: Artificial intelligence
CI: Confidence interval
DCIS: Ductal carcinoma in situ
ER: Estrogen receptor
Her2: Human epidermal growth factor receptor 2
IQR: Interquartile range
OR: Odds ratio
PR: Progesterone receptor

## Declarations

### Guarantor

The scientific guarantor of this publication is Solveig Hofvind.

### Statistics and Biometry

Two of the authors, Silje Sagstad and Marthe Larsen, have significant statistical expertise.

### Study subjects or cohorts overlap

This is a study including information from women who participated in BreastScreen Norway 2006–2019. These women are included in several other publications from BreastScreen Norway, but the specific study population used in this study has never been used for any other publication previously.

### Methodology

• retrospective

• cohort study

• multicenter study
