# Supplementary material for: Screen-detected and interval breast cancer after concordant and discordant interpretations in a population based screening program using independent double reading
Source: Eur Radiol. 2022 Apr 2;32(9):5974–85. doi: 10.1007/s00330-022-08711-9 (PMC9381607; doi:10.1007/s00330-022-08711-9)
Supplement: Supplementary file 1 — (DOCX 359 kb) [file 330_2022_8711_MOESM1_ESM.docx]

# Appendix Figure 1


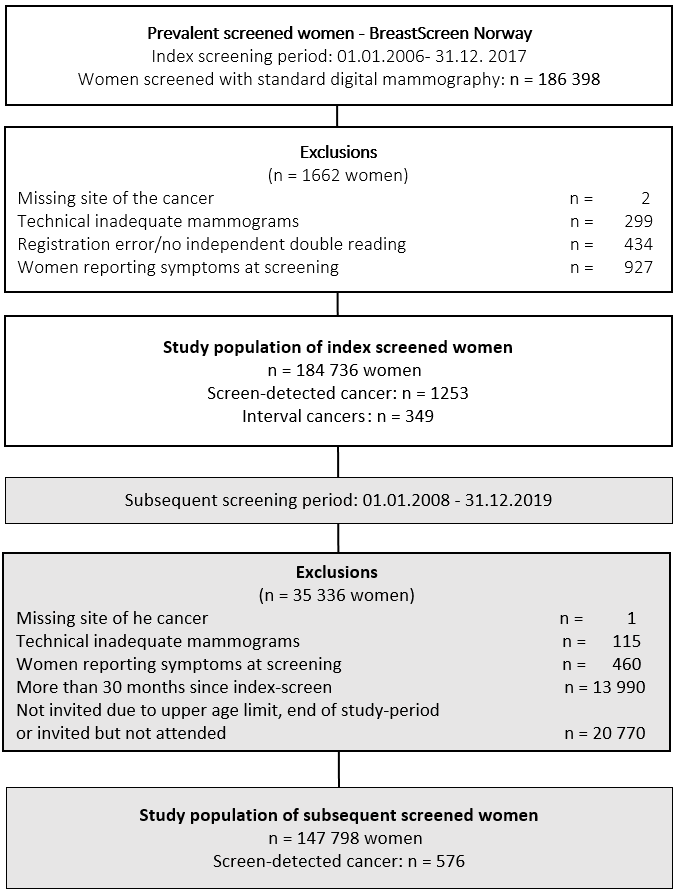


Appendix Figure 1: Flowchart of the prevalent study population. Reasons for exclusions, number of index study population and subsequent study population, number of screen-detected cancers, interval cancers and subsequent screen-detected cancer

# Appendix Figure 2


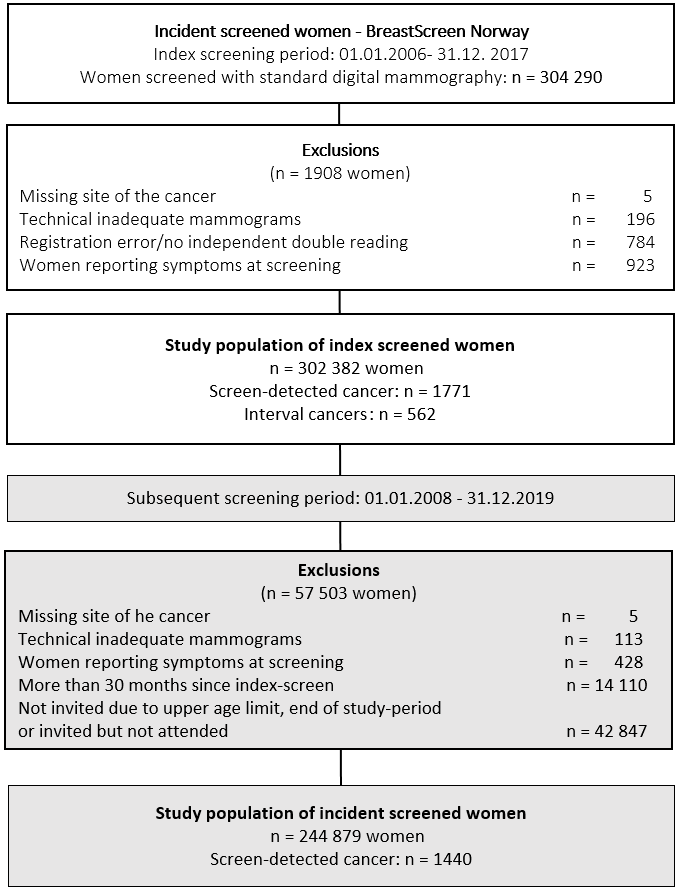


Appendix Figure 2: Flowchart of the incident study population. Reasons for exclusions, number of index study population and subsequent study population, number of screen-detected cancers, interval cancers and subsequent screen-detected cancer

# Appendix Figure 3


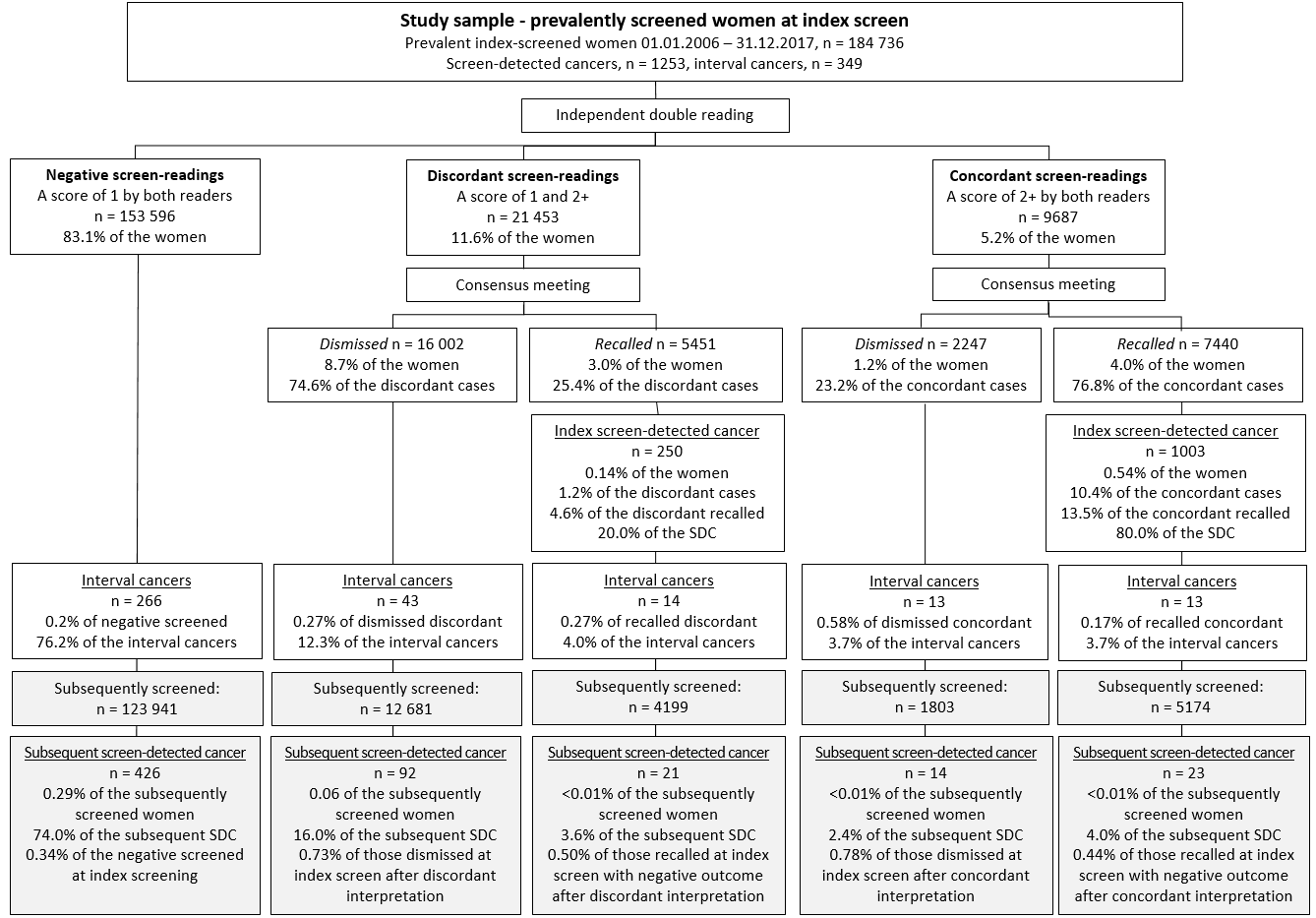


Appendix Figure 3: Flowchart of number of prevalently screening mammograms stratified by results of interpretation score at index screen and outcome of consensus. Recall rates, cancer detection rate, proportion of discordant and concordant cancers and number of interval cancers and subsequent screen-detected cancers, in a population-based screening program using independent double reading with consensus.

# Appendix Figure 4


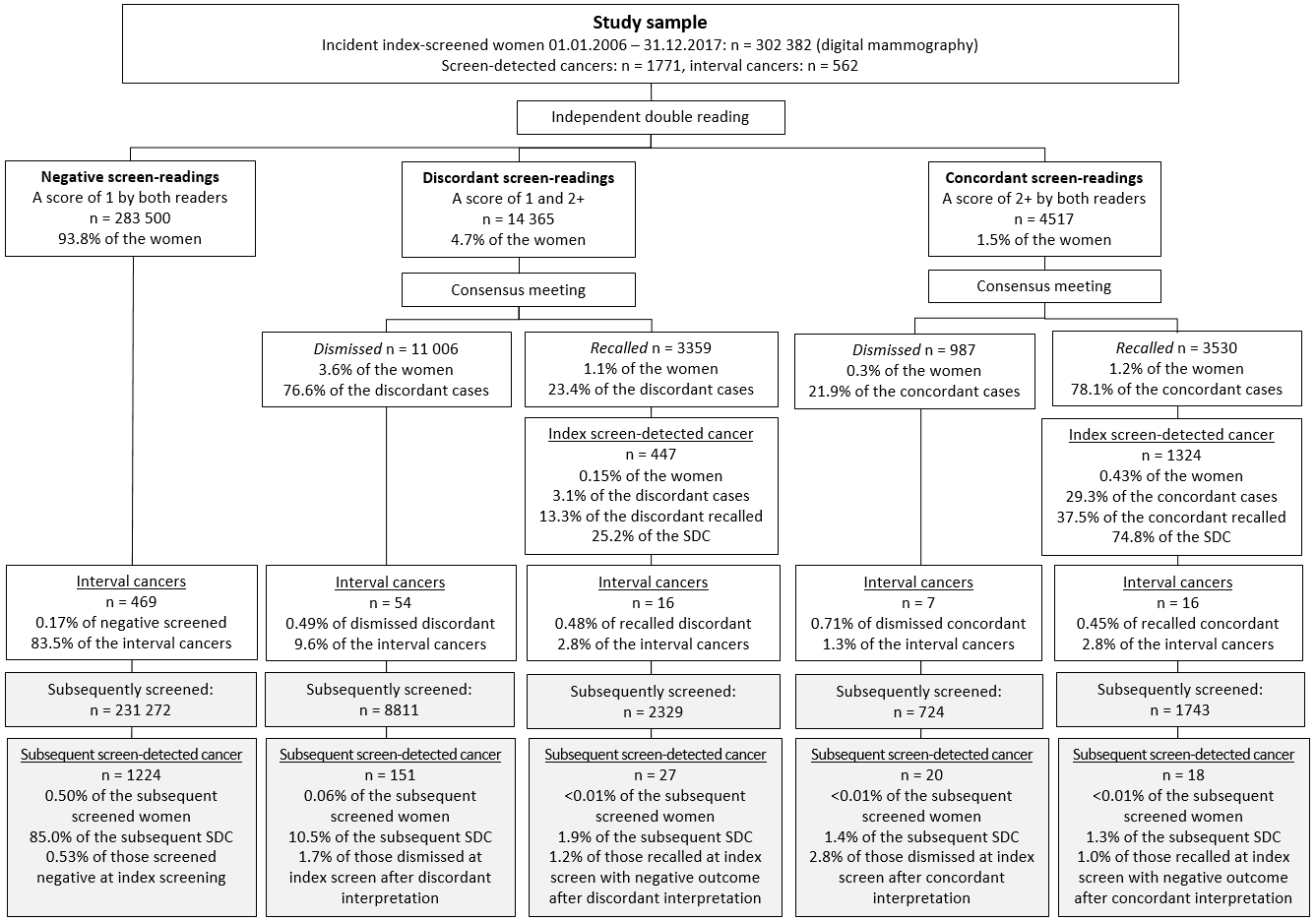


Appendix Figure 4: Flowchart of number of incident screening mammograms stratified by results of interpretation score at index screen and outcome of consensus. Recall rates, cancer detection rate, proportion of discordant and concordant cancers and number of interval cancers and subsequent screen-detected cancers, in a population-based screening program using independent double reading with consensus.
